# Objective Quantification of Bilateral Bubble Contrast Echocardiography Correlates with Systemic Oxygenation in Patients with Single Ventricle Circulation

**DOI:** 10.3390/jcdd11030084

**Published:** 2024-03-01

**Authors:** Ashley Phimister, Chana Bushee, Monica Merbach, Sai Alekha Challa, Amy Y. Pan, Andrew D. Spearman

**Affiliations:** 1 Department of Pediatrics, Division of Cardiology, Herma Heart Institute-Children’s Wisconsin, Medical College of Wisconsin, 9000 West Wisconsin Avenue, Milwaukee, WI 53226, USA; aphimister@childrenswi.org (A.P.); cbushee@mcw.edu (C.B.); mmerbach@mcw.edu (M.M.); 2 Department of Pediatrics, Division of Quantitative Health Sciences, Medical College of Wisconsin, Children’s Wisconsin, 9000 West Wisconsin Avenue, Milwaukee, WI 53226, USA; sachalla@mcw.edu (S.A.C.); apan@mcw.edu (A.Y.P.); 3 Cardiovascular Center, Medical College of Wisconsin, 8701 West Watertown Plank Road, Milwaukee, WI 53226, USA

**Keywords:** pulmonary arteriovenous malformations, bubble echocardiography, single ventricle, Glenn, Fontan, intrapulmonary shunting, hepatic factor

## Abstract

Bubble contrast echocardiography is commonly used to diagnose pulmonary arteriovenous malformations (PAVMs) in single ventricle congenital heart disease (CHD), yet previous studies inconsistently report a correlation between bubble echoes and oxygenation. In this study, we sought to re-evaluate the correlation between bubble echoes and oxygenation by assessing total bilateral shunting and unilateral shunting. We conducted a single-center, retrospective study of patients with single ventricle CHD and previous Glenn palliation who underwent a cardiac catheterization and bubble echocardiogram during the same procedure from 2011 to 2020. Spearman’s rank correlation was performed to examine the relationship between total bilateral shunting and measures of systemic oxygenation, as well as unilateral shunting and ipsilateral pulmonary vein oxygenation. For all patients (n = 72), total bilateral shunting moderately correlated with peripheral oxygen saturation (SpO_2_) (r_s_ = −0.44, *p* < 0.0001). For patients with Glenn/Kawashima circulation (n = 49), total bilateral shunting was moderately correlated (SpO_2_: r_s_ = −0.38, *p* < 0.01). In contrast, unilateral shunting did not correlate with ipsilateral pulmonary vein oxygenation for any vein measured (*p* = 0.16–*p* > 0.99). In conclusion, the total burden of bilateral bubble shunting correlated with systemic oxygenation and may better reflect the total PAVM burden from all lung segments. Unilateral correlation may be adversely influenced by non-standardized approaches to pulmonary vein sampling.

## 1. Introduction

Pulmonary arteriovenous malformations (PAVMs) are pathologic vascular connections between arteries and veins that develop in 60–100% of patients with single ventricle congenital heart disease (CHD) [1,2]. PAVMs have been recognized in single ventricle circulation for nearly 50 years but remain poorly understood [3,4,5]. PAVMs cause right to left intrapulmonary shunting and varying degrees of hypoxia. Thus, several clinical tools have been used to assess PAVMs in single ventricle circulation, including bubble contrast echocardiography.

Clinical guidelines focused on patients without CHD support that bubble echoes performed with agitated saline are highly sensitive for detecting intrapulmonary shunting (a marker for PAVMs) and have low false positive rates (<5%) [6]. Previous studies in patients with single ventricle circulation support both findings (high sensitivity and low false positive rate) [7,8,9]. Unfortunately, previous studies in patients with single ventricle circulation also reported an inconsistent correlation between unilateral bubble echo severity and measures of pulmonary vein oxygenation, including pulmonary vein saturation (SO_2_) and partial pressure of oxygen (PO_2_) [9,10,11]. Previous studies in single ventricle circulation relied on subjective assessment of shunting severity (mild, moderate, severe) or dichotomization (positive versus negative), which limited analyses to unilateral shunting and ipsilateral pulmonary vein measurements, or dichotomized comparisons with systemic oxygenation [7,8,9,10,11]. To our knowledge, no previous studies in single ventricle circulation have quantified total bilateral lung shunting and correlated with measures of oxygenation. Thus, the primary objective of this study was to evaluate the correlation between objective quantification of total bilateral lung shunting and simultaneous measures of oxygenation. As a secondary objective, we sought to evaluate the correlation between unilateral lung shunting and oxygenation, as well as investigate the association between bilateral shunting severity and clinical variables.

## 2. Materials and Methods

### 2.1. Patient Selection

We conducted a single-center, retrospective study of patients with single ventricle CHD and previous Glenn palliation who underwent a cardiac catheterization and bubble echocardiogram during the same anesthetic procedure at Children’s Wisconsin from 2011 to 2020. This period was chosen due to the availability of electronic medical records. Patients were included if they had Glenn or Fontan circulation, including Kawashima circulation (Glenn with interrupted inferior vena cava). Patients were excluded if they had 1.5 ventricle circulation (Glenn circulation with ventricular septation and antegrade pulmonary blood flow), or any native antegrade pulmonary blood flow. All catheterizations and bubble echocardiograms were performed at the discretion of the primary cardiologist and/or cardiac interventionalist. Our institutional practice is to universally obtain pre-Fontan cardiac catheterizations for surgical planning. Surveillance catheterizations are not routinely obtained post-Fontan, so all Fontan catheterizations are obtained at the request of the primary cardiologist. If patients had multiple clinical encounters with same-day catheterization and bubble study, only the first clinical encounter was included in the analysis. Data regarding patient diagnoses and clinical variables were obtained from the electronic medical record. This study was approved by the local Institutional Review Board. 

### 2.2. Bubble Echocardiograms

All bubble echocardiograms were performed using transthoracic echocardiography and agitated saline injection into one or both branch pulmonary arteries during cardiac catheterizations. We assessed the severity of intrapulmonary shunting identified by bubble echo with independent methods: (1) subjective quantification, and (2) objective quantification. We performed objective quantification of shunting to utilize a continuous variable that allowed summation of bilateral shunting and subsequent correlation with systemic oxygenation, rather than exclusive assessment of unilateral lung shunting with a subjective/semi-quantitative approach.

Subjective quantification was performed similarly to previously published methods by an Attending pediatric cardiologist (ADS) based on bubble density in the common atrium (percent opacification of the chamber): negative (no identified bubbles), trivial (<5%), mild (5–25%), mild-moderate (25–50%), moderate (50–75%), moderate-severe (75–95%), and severe (>95%) [6,9,12,13]. 

**Figure 1:**
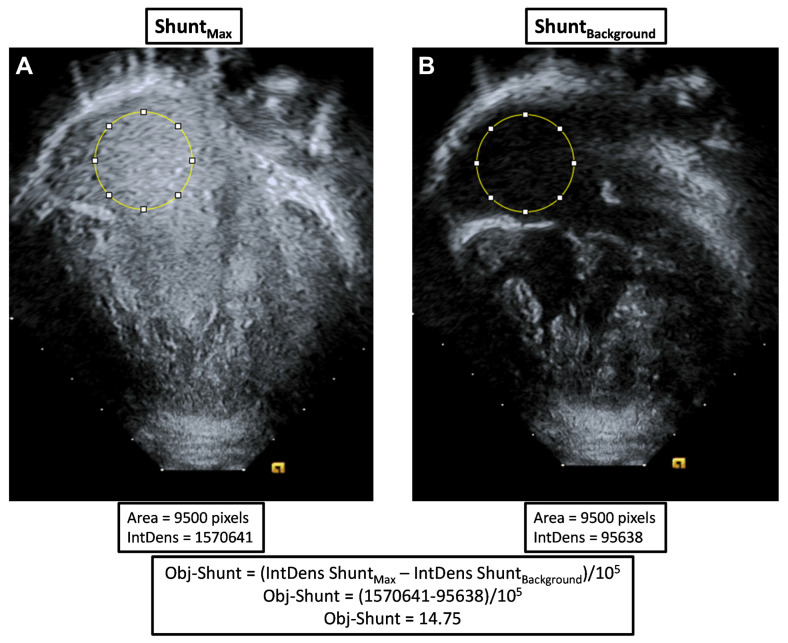
Example images of objective quantification of bubble echo shunting severity. (**A**) A still frame image captured at the time of maximal bubble shunting intensity (Shunt_Max_) of a patient with severe shunting in the right lung. (**B**) A still frame image captured prior to agitated saline injection (Shunt_Background_). Using ImageJ, we measured the integrated density (IntDens) of the same region of interest (yellow circle in the common atrium) to quantify the pixel intensity of that region. Objective shunting (Obj-Shunt) was determined by calculating the difference between Shunt_Max_ and Shunt_Background_ and dividing by 10^5^.

Objective quantification was performed by a blinded reviewer (MM) using ImageJ (version 1.53t), similar to a previously published method [14]. Bubble echo clips were downloaded and analyzed offline with a unique study identification number. Three still frame images were captured prior to shunting and averaged to measure the background signal (Shunt_Background_). Measurement of background signal is necessary to account for differences in acoustic windows. One still frame image was captured at the time of maximal bubble shunting intensity (Shunt_Max_). Using the same region of interest within the common atrium (same position and size), we measured the Integrated Density of Shunt_Background_ and Shunt_Max_, which quantifies the pixel intensity in the region of interest. Objective shunting (Obj-Shunt) for each bubble study was then calculated as shown below and illustrated with an Equation (1) and an example in Figure 1.

Obj-Shunt = (Integrated Density Shunt_Max_ − Integrated Density Shunt_Background_)/10^5^ (1)

We quantified shunting in individual lungs and the total bilateral lung shunting: left lung objective shunting (Obj-Shunt_Left_), right lung objective shunting (Obj-Shunt_Right_), and total bilateral objective shunting (Obj-Shunt_Total_ = Obj-Shunt_Left_ + Obj-Shunt_Right_).

### 2.3. Measures of Oxygenation

Measures of oxygenation were collected from clinically available data obtained during cardiac catheterization. Oxygenation variables included peripheral pulse oximetry (SpO_2_), descending aorta saturation (DAo SO_2_), descending aorta partial pressure of oxygen (DAo PO_2_), pulmonary vein saturation (PV SO_2_), and pulmonary vein partial pressure of oxygen (PV PO_2_). Collection of oxygenation data was at the discretion of the clinical providers and results were recorded when available. 

### 2.4. Statistical Analysis

Cohort data are expressed as median [interquartile range] for continuous data and n (%) for categorical data unless otherwise stated. Spearman’s rank correlation coefficient (r_s_) was calculated to assess the relationship between subjective and objective assessment of bubble echo shunting, as well as the relationship among Obj-Shunt_Total_, Obj-Shunt_Left_, Obj-Shunt_Right_, and measures of oxygenation obtained during simultaneous cardiac catheterization. Correlation strength was based on published guidelines: 0–0.3 indicates weak correlation, 0.3–0.7 indicates moderate correlation, and 0.7–1 indicates strong correlation [15]. To evaluate the differences in Obj-Shunt_Total_ by clinical variables (sex, primary cardiac diagnosis, heterotaxy diagnosis, interrupted inferior vena cava (IVC), bilateral bidirectional Glenn, and major blood group type), we performed a Mann–Whitney–Wilcoxon test or Kruskal–Wallis test. A *p*-value < 0.05 was considered statistically significant. Analyses were performed using SAS 9.4 (SAS Institute, Cary, NC, USA), SPSS 26.0 (IBM Corp., Armonk, NY, USA), and GraphPad Prism 10 (GraphPad Software, San Diego, CA, USA).

## 3. Results

### 3.1. Patient Characteristics

A total of 72 patients with single ventricle CHD were included in this study (Table 1). Most patients had Glenn circulation (41/72, 57%), but a significant number had Kawashima (8/72, 11%) or Fontan circulation (23/72, 32%). Time from surgery to catheterization was not different between the Glenn and Kawashima groups (duration post-Glenn: 1.9 [1.2, 2.9] years; duration post-Kawashima: 2.6 [1.7, 3.6] years; *p* = 0.17), so subsequent analyses combined the Glenn and Kawashima sub-groups. Most patients in the Glenn cohort had at least one pulmonary vein blood gas sample recorded (30/41, 71%); however, no patients in the Kawashima cohort had a pulmonary vein blood gas sample (0/8, 0%). Patient characteristics for the entire cohort and sub-groups are summarized in Table 1.

### 3.2. Objective Quantification of Bubble Echocardiograms

**Figure 2:**
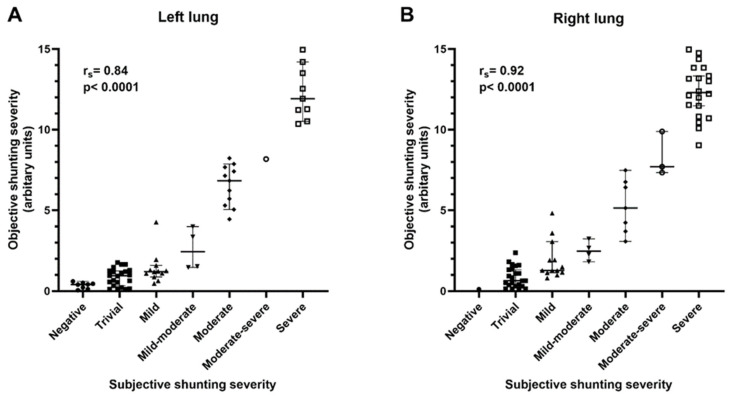
Positive correlation between semi-quantitative subjective assessment of bubble echo severity and quantitative objective assessment of bubble echo severity. Scatter plots with median and 95% confidence intervals show data for bubble studies performed in both the (**A**) left (n = 67, r_s_ = 0.84, *p* < 0.0001) and (**B**) right (n = 71, r_s_ = 0.92, *p* < 0.0001) lungs.

Among all 72 patients, 71 patients had a bubble echo performed in the right lung and 67 patients had a bubble echo performed in the left lung. There was a strong positive correlation between subjective and objective quantification of bubble echo shunting (right lung: r_s_ = 0.92, *p* < 0.0001; left lung: r_s_ = 0.84, *p* < 0.0001) (Figure 2). There were 8/72 (11.1%) patients with a negative bubble echo unilaterally, but only 1/72 (1.4%) patients had a negative bubble echo bilaterally. Most patients with a negative bubble study had Fontan circulation (6/8, 75%) and Fontan palliation performed a median of 15.3 years prior to the catheterization/bubble echo. The one patient with a negative bubble echo bilaterally had Fontan palliation 17.5 years prior to the catheterization/bubble echo.

### 3.3. Correlation of Total Bilateral Intrapulmonary Shunting and Systemic Oxygenation

**Figure 3:**
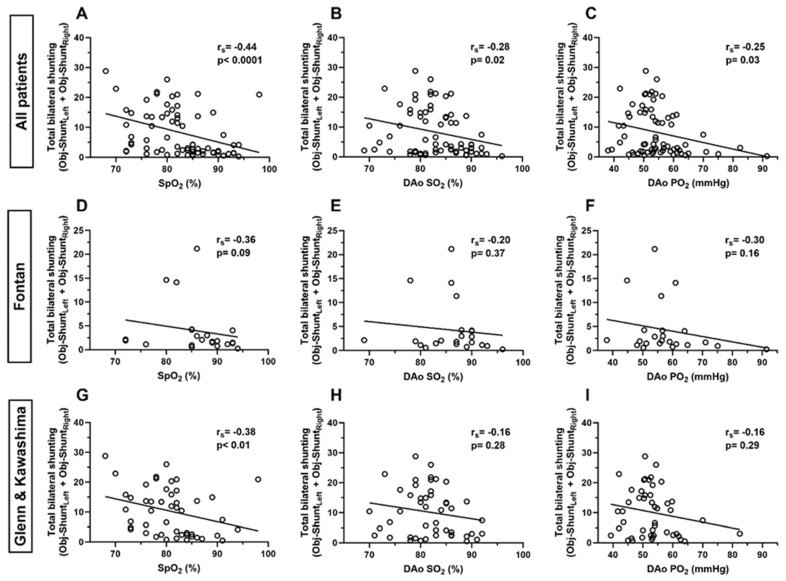
Correlations between total bilateral intrapulmonary shunting and systemic oxygenation measurements. Figures show XY scatter plots with linear best-fit slopes for (**A**–**C**) all patients (n = 72), (**D**–**F**) patients with Fontan circulation during testing (n = 23), and (**G**–**I**) patients with Glenn or Kawashima circulation during testing (n = 49). r_s_ indicates Spearman’s rank correlation coefficient. SpO_2_—peripheral oxygen saturation, DAo SO_2_—descending aorta oxygen saturation, DAo PO_2_—descending aorta partial pressure of oxygen.

Among the entire cohort, there was a moderate negative correlation between total bilateral shunting (Obj-Shunt_Total_) and SpO_2_ (r_s_ = −0.44, *p* < 0.0001), a weak negative correlation between Obj-Shunt_Total_ and DAo SO_2_ (r_s_ = −0.28, *p* = 0.02), and a weak negative correlation between Obj-Shunt_Total_ and DAo PO_2_ (r_s_ = −0.25, *p* = 0.03) (Figure 3).

Among patients with Glenn and Kawashima circulation, there was a moderate negative correlation between Obj-Shunt_Total_ and SpO_2_ (r_s_ = −0.38, *p* < 0.01), but there was no significant correlation between Obj-Shunt_Total_ and DAo SO_2_ (r_s_ = −0.16, *p* = 0.28) or DAo PO_2_ (r_s_ = −0.16, *p* = 0.29). Additionally, there was no significant correlation between Obj-Shunt_Total_ and any measure of systemic oxygenation in patients with Fontan circulation (Figure 3).

### 3.4. Correlation of Unilateral Intrapulmonary Shunting and Pulmonary Vein Oxygenation

Among patients in the Glenn cohort, there was no significant correlation between unilateral intrapulmonary shunting (Obj-Shunt_Left_ or Obj-Shunt_Right_) and any measure of pulmonary vein oxygenation; however, these analyses are likely underpowered given the limited number of patients with pulmonary vein blood gas data available (Table 2). 

The small sample size also limited the comparison of shunting with simultaneous upper pulmonary vein and lower pulmonary vein oxygenation. However, Table 3 demonstrates pulmonary vein oxygenation data and shunting quantification data from patients with simultaneous upper and lower pulmonary vein data. Patient #1 (Glenn-1) had trivial intrapulmonary shunting in both lungs and showed similar oxygenation in the bilateral lower and upper pulmonary veins. In contrast, patient #2 had severe shunting in both lungs (Obj-shunt > 10) and showed decreased oxygenation in the bilateral lower pulmonary veins (41 and 43 mmHg) compared to the left upper pulmonary vein (229 mmHg). Similarly, patient #3 showed similar oxygenation in the left upper (146 mmHg) and left lower (122 mmHg) pulmonary veins where bubble echo demonstrated moderate shunting, but there was a discrepancy between the upper (138 mmHg) and lower (55 mmHg) right-sided veins where the bubble echo demonstrated severe shunting. Finally, patient #4 was able to increase oxygenation in the left upper pulmonary vein with supplemental oxygenation (437 mmHg; trivial left-sided shunting), was able to increase oxygenation in the right upper pulmonary vein with supplemental oxygen (461 mmHg; severe right-sided shunting), but was not able to increase oxygenation in the right lower pulmonary vein with supplemental oxygen (106 mmHg; severe right-sided shunting). These data suggest that the lower lung segments may be more impacted by PAVMs and thus the lower pulmonary veins may be more profoundly hypoxemic than the upper pulmonary veins.

### 3.5. Relationship between Total Intrapulmonary Shunting and Clinical Variables 

At the time of clinical testing, those patients with Glenn or Kawashima circulation had greater total bilateral shunting (Obj-Shunt_Total_) than patients with Fontan circulation (*p* < 0.01 for both; Supplemental Figure S1). Among the entire cohort, total bilateral shunting was significantly higher in patients with a history of interrupted IVC compared to those without (median 14.1 vs. 3.5, *p* < 0.01) (Table 4). No other clinical variables in the total cohort were associated with total bilateral shunting burden. Among the sub-groups of patients with Fontan circulation or Glenn/Kawashima circulation at the time of testing, no clinical variables were associated with the total bilateral shunting burden; however, history of an interrupted IVC trended toward statistical significance (*p* = 0.08 for both sub-groups). 

Among the sub-group of patients with Fontan circulation, who underwent their catheterization and bubble echo a median of 11.7 years after Fontan palliation, there was a moderate negative correlation between total bilateral shunting and duration post-Fontan (r_s_ = −0.46, *p* = 0.02), indicating that shunting decreases post-Fontan after incorporating hepatic vein blood into pulmonary blood flow. Conversely, among the sub-group of patients with Glenn and Kawashima circulation, there was no significant correlation between total bilateral shunting and duration post-Glenn/Kawashima (r_s_ = −0.04, *p* = 0.80), potentially due to the short median duration after Glenn and Kawashima palliation (1.9 [1.2, 2.9] years and 2.6 [1.7, 3.6] years, respectively).

Among all patients and those with Glenn circulation, there was a moderate negative correlation between age at Glenn palliation and total bilateral shunting (all patients: r_s_ = −0.30, *p* < 0.01; Glenn patients: r_s_ = −0.34, *p* = 0.03). However, among those patients with Fontan circulation and previous Glenn palliation, there was no significant correlation between age at Glenn palliation and total bilateral shunting (r_s_ = −0.26, *p* = 0.19).

## 4. Discussion

In this study, our data demonstrate that an individual’s total bilateral burden of bubble shunting correlated with systemic oxygenation, including non-invasive peripheral saturation (SpO_2_) and invasive descending aorta measures of oxygenation (DAo SO_2_ and DAo PO_2_). In order to quantify an individual’s total bilateral burden of intrapulmonary shunting, we report the novel application of objective quantification of bubble echo shunting in patients with single ventricle circulation. 

Kroon et al. previously reported the utility of objectively quantifying bubble echo shunting in patients with hereditary hemorrhagic telangiectasia (HHT) [14]. Similar to our study, they reported a strong correlation between objective quantification and standard subjective semi-quantitative assessment (n = 465, r_s_ = 0.89, *p* < 0.0001). As shown in Figure 2, our blinded objective quantification had excellent agreement with subjective assessment. However, the benefit of objective quantification is that unilateral shunting severity can easily be summed to quantify total bilateral shunting severity, which allows comparison with systemic variables (i.e., SpO_2_).

Multiple previous studies compared bubble echoes with various measures of oxygenation, including initial studies that dichotomized bubble echo results (positive versus negative) and more recent studies that assessed the correlation of unilateral shunting with pulmonary vein oxygenation [7,8,9,10,11]. As expected, initial studies reported that individuals with positive bubble studies had statistically decreased arterial saturations at rest (88% vs. 95%, *p* < 0.01) and with exercise (78% vs. 89%, *p* = 0.01) or trended towards lower saturations at rest (81 vs. 88%, *p* = 0.10) and higher Hgb (16.4 vs. 14.7 g/dl, *p* = 0.18) compared to those with negative bubble studies [7,8]. In the first study assessing the relationship between bubble shunting and pulmonary vein oxygenation, Feinstein et al. reported that only 26% of lungs with a positive bubble study had pulmonary vein desaturation (defined as saturation < 94% and using the lowest available saturation if multiple veins were sampled) [9]. More recently, Asada et al. reported no correlation between unilateral bubble echo severity and ipsilateral pulmonary capillary wedge saturation (as a surrogate for pulmonary vein saturation) in a larger cohort of patients (n = 140) with Fontan circulation (right side, *p* = 0.34; left side, *p* = 0.85) [10]. Finally, in the most recent study, Kartik et al. reported a moderate correlation between the degree of pulmonary vein desaturation and ipsilateral bubble echo severity (r_s_ = 0.58, *p* < 0.001), albeit with a relatively small cohort (n = 25 with Glenn circulation) [11]. They described that they averaged samples from both the upper and lower pulmonary veins. Interestingly, their cohort was somewhat older (8.8 ± 3.4 years) for patients with Glenn circulation, which may suggest that their cohort had more advanced PAVM remodeling than typical patients with Glenn circulation prior to Fontan palliation. In all studies, there were no direct comparisons between total bilateral shunting and systemic oxygenation, nor were there comparisons between upper and lower pulmonary vein oxygenation.

Similarly, our data show that unilateral bubble echo shunting poorly correlates with an ipsilateral pulmonary vein blood gas sample (Table 2). Patient data in Table 3 illustrate that oxygenation differences may exist between upper pulmonary veins and lower pulmonary veins in patients with pronounced intrapulmonary shunting. It is plausible that a single pulmonary vein sample reflecting a single lobe does not accurately reflect the entire lung physiology that is captured with a unilateral bubble echo. We speculate that greater aortopulmonary collateral flow to upper lobe segments may preferentially increase oxygenation in the upper lobes, whereas intrapulmonary shunting may predominate in the lower lobes and thus preferentially decrease oxygenation in the lower lobes. This is in line with findings by Kartik et al. who reported a greater PAVM burden in the lower lung zones in patients with Glenn circulation [11]. Unfortunately, our study was limited by the number of patients with both upper and lower pulmonary vein samples, so we are unable to definitively assess this question.

Finally, in line with other studies, we re-demonstrated that intrapulmonary shunting is nearly universal in patients with palliated single ventricle circulation [2,10]. Only one patient in our cohort had a negative bubble study bilaterally, and this individual had Fontan palliation >17 years prior to the bubble study. This further supports that the re-incorporation of hepatic vein blood into pulmonary blood flow can resolve PAVMs [16,17,18,19]. Interestingly, we also observed that earlier age at Glenn palliation was correlated with greater bilateral shunting. This suggests that there may be a developmental vulnerability of the lungs that predisposes to PAVM formation after Glenn palliation.

Our study has several limitations that are inherent in a single-institution retrospective study. Despite being larger than most other similar studies, our sample size is relatively small with multiple inter-related clinical variables. Additionally, multiple clinical providers performed bubble echoes without a standardized approach for the volume of agitated saline per injection, which may lead to differences in bubble echo severity based on technique. A standardized approach (volume of saline and air, or even volume of saline and air-adjusted for body size and/or pulmonary blood flow) within our institution and across all pediatric cardiac catheterization laboratories may help improve the interpretation and correlation of bubble echoes. Lastly, there was likely selection bias in which patients had bubble echoes performed. Many patients with single ventricle circulation never had a simultaneous catheterization and bubble study, so it is unknown if our data are generalizable to all patients with single ventricle circulation or if the correlation may be stronger with a more systematic approach.

## 5. Conclusions

In conclusion, an individual’s total burden of bubble shunting, as assessed with bilateral objective quantification of bubble shunting, correlated with systemic oxygen saturation. Unilateral bubble echo shunting did not correlate with pulmonary vein oxygenation. Additional research is needed to determine if unilateral correlation can be improved with a standardized approach for performing bubble echoes and sampling pulmonary vein blood. Greater standardization is critical for multi-institution collaboration, clinical trials, and ultimately developing new clinical interventions for PAVMs.

## Figures and Tables

**Table 1:** Cohort demographics.

Cohort Demographics		*n* = 72
Age (years)		12.5 [9.8, 19.1]
Male sex		41 (56.9)
Primary cardiac diagnosis	AVSD	19 (26.4)
	DILV	12 (16.7)
	DORV	9 (12.5)
	HLHS	27 (37.5)
	PA IVS	1 (1.4)
	TA	4 (5.5)
Heterotaxy		26 (36.1)
Interrupted IVC		13 (18.1)
Bilateral Glenn		15 (20.8)
Major blood group	A	21 (29.1)
	B	12 (16.7)
	AB	1 (1.4)
	O	38 (52.8)
Circulatory stage at catheterization	Glenn	41
	Kawashima	8
	Fontan	23
Duration post-Glenn (years) ^#^		1.9 [1.2, 2.9]
Duration post-Kawashima (years) ^$^		2.6 [1.7, 3.6]
Duration post-Fontan (years) ^^^		11.7 [5.2, 15.8]
Any pulmonary vein data obtained (Glenn circulation only)		30 (71.4)

^#^ n = 41, ^$^ n = 8, ^^^ n = 23. No statistically significant difference between the duration from Glenn palliation to catheterization or the duration from Kawashima palliation to catheterization (*p* = 0.17, Mann–Whitney–Wilcoxon test). AVSD—atrioventricular septal defect, DILV—double inlet left ventricle, DORV—double outlet right ventricle, HLHS—hypoplastic left heart syndrome, IVC—inferior vena cava, PA IVS—pulmonary atresia intact ventricular septum, TA—tricuspid atresia.

**Table 2:** Correlation between unilateral intrapulmonary shunting and oxygen measurements in the ipsilateral pulmonary vein.

Cohort	Oxygen Measurement	Number of Measurements	Correlation Coefficient (r_s_)	*p*-Value
Glenn	LUPV SO_2_	17	0.00	>0.99
	LUPV PO_2_	16	−0.05	0.86
	RUPV SO_2_	19	0.07	0.76
	RUPV PO_2_	18	0.07	0.78
	LLPV SO_2_	11	−0.04	0.91
	LLPV PO_2_	11	−0.20	0.56
	RLPV SO_2_	9	−0.47	0.21
	RLPV PO_2_	8	−0.55	0.16

Glenn cohort n = 41. LUPV—left upper pulmonary vein, RUPV—right upper pulmonary vein, LLPV—left lower pulmonary vein, RLPV—right lower pulmonary vein, SO_2_—oxygen saturation (%), PO_2_—partial pressure of oxygen (mmHg).

**Table 3:** Oxygenation differences between upper and lower pulmonary vein samples.

Patient	Objective Shunting Left	Objective Shunting Right	Inhaled Supplemental O_2_	LUPV	LLPV	RUPV	RLPV
Glenn-1	1.30	1.14	21%	124 mmHg	156 mmHg	121 mmHg	93 mmHg
				99%	99%	99%	96%
			-	-	-	-	-
				-	-	-	-
Glenn-2	10.52	12.38	35%	229 mmHg	41 mmHg	-	43 mmHg
				99%	71%	-	75%
			-	-	-	-	-
				-	-	-	-
Glenn-3	7.41	13.84	25%	146 mmHg	122 mmHg	138 mmHg	55 mmHg
				99%	98%	98%	86%
			-	-	-	-	-
				-	-	-	-
Glenn-4	0.34	13.33	21%	96 mmHg	-	-	-
				99%	-	-	-
			100%	437 mmHg	-	461 mmHg	106 mmHg
				100%	-	100%	96%

LUPV—left upper pulmonary vein, RUPV—right upper pulmonary vein, LLPV—left lower pulmonary vein, RLPV—right lower pulmonary vein, O_2_—oxygen.

**Table 4:** Association between total intrapulmonary shunting and categorical clinical variables.

Cohort	Clinical Variable	*p*-Value
All patients (n = 72)	Sex	0.43
	Primary cardiac diagnosis	0.79
	Heterotaxy	0.49
	Interrupted IVC	<0.01
	Bilateral BDG	0.69
	Major blood group	0.88
Fontan (n = 23)	Sex	0.74
	Primary cardiac diagnosis	0.44
	Heterotaxy	0.60
	Interrupted IVC	0.08
	Bilateral BDG	0.66
	Major blood group	0.74
Glenn + Kawashima (n = 49)	Sex	0.35
	Primary cardiac diagnosis	0.36
	Heterotaxy	0.93
	Interrupted IVC	0.08
	Bilateral BDG	0.78
	Major blood group	0.51

BDG—bidirectional Glenn, IVC—inferior vena cava.

## Data Availability

All data are available upon request.

## Supplementary Materials

The following supporting information can be downloaded at: https://www.mdpi.com/article/10.3390/jcdd11030084/s1, Figure S1: Comparison of total bilateral intrapulmonary shunting among patients at different stages of single ventricle circulation.
