# Supplementary material for: Objective Quantification of Bilateral Bubble Contrast Echocardiography Correlates with Systemic Oxygenation in Patients with Single Ventricle Circulation
Source: J Cardiovasc Dev Dis. 2024 Mar 1;11(3):84. doi: 10.3390/jcdd11030084 (PMC10971280; doi:10.3390/jcdd11030084)
Supplement: Supplementary file 1 [file jcdd-11-00084-s001.zip › jcdd-2790322-supplementary.pdf]

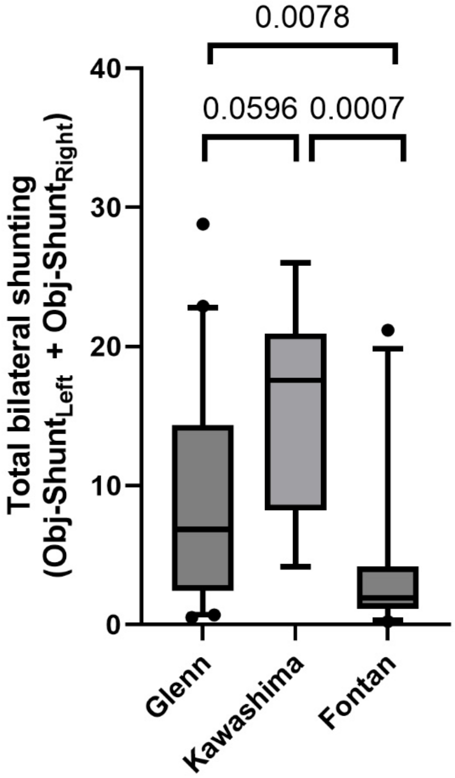

Supplemental Figure S1. Comparison of total bilateral intrapulmonary shunting among patients at different stages of single ventricle circulation. Graph demonstrates a box-and-whisker plot with median and interquartile ranges (box), as well as 5th-95th percentiles (whiskers) and single points outside the 5th-95th percentiles. Analyzed using Kruskal Wallis test with Dwass, Steel, Critchlow-Fligner method for multiple comparison adjustment.
